# The Universals of Games and Sports

**DOI:** 10.3389/fpsyg.2020.593877

**Published:** 2020-10-21

**Authors:** Pierre Parlebas

**Affiliations:** ^1^Professor Emeritus at University Paris Descartes, Paris, France; ^2^Honorary Doctor University of Lleida, Catalonia, Spain; ^3^Honorary Doctor Universidade Estadual de Campinas, São Paulo, Brazil

**Keywords:** motor action, traditional sporting game, internal logic, motor praxeology, structural invariance, sociometric assessment, ethnoludism

## Abstract

The so-called *traditional* motor games are group situations that function like small-scale societies, full of emotionally rich vicissitudes and proper objectives, alliances, and antagonisms. Traditional games have certainly been the object of many dispersed, really interesting studies, but no general conception of them, based on a scientifically supported methodological approach, has been developed so far. How do these games work? Does their development depend on sheer chance? Does it respond to any underlying structures? Is this development anyhow related to the socio-emotional dynamics of the group of players? As a whole, do these games, so different from each other, have any common characteristics that generate similar effects on the personality of the players? In the end, is what we know about a given game comparable and generalisable to any other one?

## In Search of a General, Theoretical Framework

We search for a general theoretical framework that bring together, in a coherent and reasonable way, the whole set of traditional games and sports, whatever their area of reference may be. Can little terroirs’ local attachment be harmonious with global, worldwide perspectives? Major international organisations insist on the positive role of sporting games in favour of entente and peace between peoples, but can this be as solid a venture as proclaimed when everybody can notice the weight and intensity of those acts of rivalry that abound in games?

### An Ambiguous Ludomotricity

A simple examination of the studies produced in many countries brings to light the extreme variety of traditional games, which, while abiding to the cultural norms of their context, exhibit original characteristics that sometimes are antipodal to each other. Certainly, if we are not careful, traditional games can become a ground for discord rather than a ground for concord: *ludodiversity* can also provoke cultural, political pressure in favour of nationalist withdraws likely to increase hostility toward the others.

Many recreational sports practices exacerbate the impulse for belligerence and domination of the others, what hardly seems to be a school of solidarity and understanding between peoples. Competitive antagonism and cultural discrepancies: Are traditional games condemned to inevitably exacerbate hostility between players on the one hand, and between cultural communities on the other? In this sense, what sort of findings and analyses can inform us about the specific nature of motor games, about their internal reality? That is, can we aspire to detect any kind of unity behind their immense variety?

### An Approach Centred on the Motor Action

The systematic observation and analysis of field, pertinent data, carried out in collaboration with researchers of different nationalities, has allowed us to develop new methodological approaches adapted to the study of motor games. Our general purpose has always been twofold:

• *To develop tools and methodological approaches* properly connected with ludomotor practices as to reveal phenomena specific to the motor action deployed during games playing, defining new concepts when necessary and taking into account the positive and negative effects exerted on the participants.
• *To synthesise these different results*, for we cannot feel satisfied with small pieces of research, monographs or isolated experimental data, no matter how important these may be. It seems interesting to conceive a general theory of motor games that place them within the great cultural creations, leading to more or less profound consequences in the social, educational, and political fields.

As usual, these two perspectives can only be successfully developed if they are called upon in constant interaction, because they feed off each other. For instance, an examination of all the motor practices reveals a split-up between two sectors. We can distinguish two fundamental domains in ludomotricity, quite an obvious distinction apparently which seems far from having been fully identified:

• *The domain of psychomotor situations* including situations that require a single actor and which therefore do not allow any relevant motor interaction with anyone else –which does not prevent the presence of other people, spectators for example.
• *The domain of sociomotor situations* including situations that take their reality only through operative motor interactions between several participants.

The critical point of our theoretical framework lies in the choice of one certain scientific specificity, our intention being to base the analysis on the contents and forms of motor action itself as revealed during the practice of physical activities and sports. The interdisciplinary aspect of such a praxeological approach is obvious, but it must always be centred on the motor conducts of persons in total dependence on their cultural expression.

### Confusions to Avoid

The first trap to avoid is the confusion between “traditional games” and “sports.” Of course, they have much in common: they all are based on a motor action subject to a system of competition rules which determines its internal logic. However, a major difference separates them: some of these motor situations have been chosen and intensely promoted by international institutions that have shaped them in the image of their socio-economic universe. In fact, only those motor games best adapted to the demands of a certain kind of mass spectacle, favouring competition and the consecration of an elite of winners, have been retained. This is what is called *sport*, which is based on the simultaneous presence of four necessary and sufficient distinctive features: motricity, rules, competition, and institution. *Sport is the set of motor situations codified in the form of competition and institutionalised.* The nomenclature problem is not neutral. We thus fundamentally differentiate “sport” from “traditional games,” although we will use the expressions *motor games* or *sporting games* when these two sectors are to be considered together.

Sport is opposed to *non-sport*. The main consequence of this fracture underlines that those activities which do not subscribe to the criteria of the sports spectacle –that is to say hundreds of traditional games– will be excluded from the field of valued practices, and from the field of research as an insidious consequence! It is also astounding that the aforementioned institutional dimension makes institutionalisation itself invisible. Is such an exclusion of traditional games from the sphere of noble activities desirable, therefore considered to be natural and taken for granted? Can scientific research accept to bow down before any received ideas, or must it break up with the categories of thought imposed by sports institutions? Can we try to highlight, alongside the indisputable contributions of sport, the specific and differential resources of traditional games?

## The Presence of Universals

Thanks to many contributions carried out for several decades, it has been revealed possible to detect strong regularities in the ludic functioning behind the immense variety and apparent confusion of the data collected in the field. As Propp (1970) clearly showed in his study of fairy tales, Lévi-Strauss (1987) in the phenomena of kinship, or Chomsky (1971) in the analysis of language, deep structures exist under the surface of apparent events. Even more, as Lévi-Strauss (1983) stated, it is more than advisable to “discover the laws of order underlying the observable diversity”: Behind the superficial disorder that is all the rage in traditional games, there is an in-depth order in there too. We call “universals” these *laws of order*, these underlying objective systems on top of which the praxic exchanges that can be observed in all games and in all sports are built: operational models which represent the basic structures of the functioning of any sporting game, bearers of the fundamental features of its internal logic.

In any game and in any sport we can for example identify the “network of motor interactions” which formalises all the operational motor communications permitted by the rules. This objective model is the irrefutable canvas for any relevant motor exchanges in any sporting game (mutual aid or antagonism), whether it is football or Prisoners’ base, volleyball or Dodgeball. This universal represents the equivalence class of the communication networks of all sports games, whichever they are. It is a class-invariant that shows many concrete potentialities. Within this equivalence class the actual form can indeed vary from game to game, but always keeping its identity as a network of motor interactions based on relations of *solidarity* and *rivalry*. For any specific game or sport, only one network of this class is accredited: the network of motor interactions associated with each game is therefore a strict invariant.

In this sense, any universal has two planes of understanding: the strict level of each game in particular, where it is a unique invariant, and the level of the set of games in general, where it represents a generative invariant, that is to say an equivalence class capable of generating the potential structure of each game. In other words, the universals of each game are a *species* of a higher level *genre* which encompasses all the particular universals.

After a morphological study of motor games centred on their essential, dynamic resources, we have been able to identify seven universals: the network of motor interactions, the network of scoring interactions, the scores system, the graph of transitions of sociomotor roles, the graph of changes in sub-roles, the gestemic system, and the praxemic system. These models are not independent of each other. Quite on the contrary, their respective characteristics are frequently inter-influenced. Thus, for instance, the network of changes of roles is directly linked to the network of interactions in many games such as the Hawk, the Bear and its keeper, the *Galine*. In order to illustrate the objective resources of these generative structures we propose to present below, in broad outline, one of these generative structures and to suggest its explanatory scope.

## The Network of Motor Interactions

This universal represents the virtual canvas on which all ludic exchanges develop. It is undoubtedly the element where everything is to be played out according to the constraints of the internal logic, such as the ways of acting and communicating. Each sporting game stages its own universal leading to extremely varied particular behaviours, very rich in relational consequences: The universal of team sports shows a great relational clarity that makes it a reference of undeniable interest while giving us the opportunity to compare with it the networks of traditional games.

### An Exemplary Network

**FIGURE 1:**
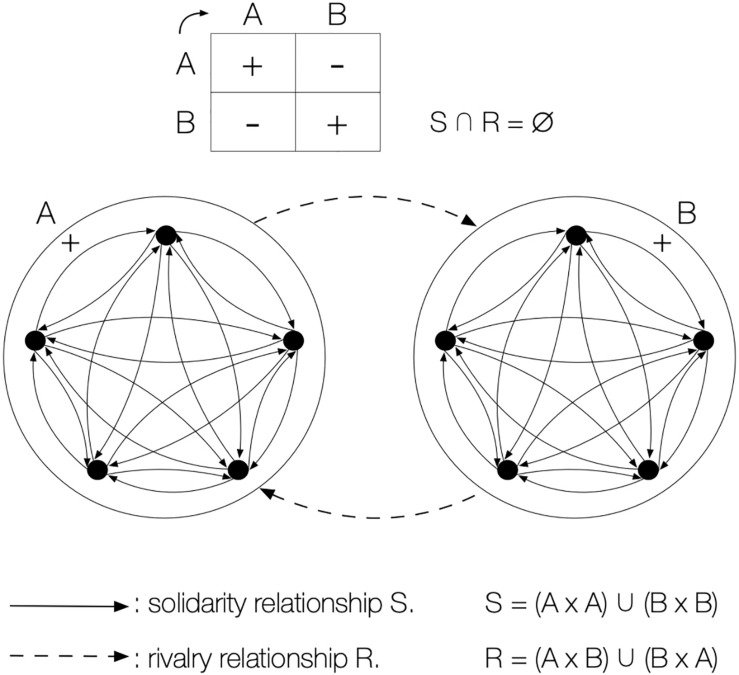
Basketball’s universal of motor interactions. Independently of the number of players, this model represents the universal of motor interactions of all European team sports. It is a symmetric duel of teams that strictly opposes two cliques whose relationships of cooperation and opposition are exclusive and stable.

As an example, we are going to examine the basketball’s model (Figure 1), which represents the smallest expression of all the motor interaction networks of classic team sports: e.g., football, handball, hockey, water polo. By “network of motor interactions” we mean the graph whose vertices are players and whose arcs represent the operational interactions authorised by the rules: passing, tackling, shooting, stealing, etc. This network is considered *stable* because rivalry and solidarity relationships between players remain constant throughout the game, and it is said to be *exclusive* because two participants can never be simultaneously partners and opponents, a principle contradicted by the so-called *paradoxical* games as we will have the chance to prove.

In this case, this graph is kind of a sketch: it is the canonical image of total opposition between two identical, strongly united clans that imposes itself as a “duel of teams.” When the two opposing teams have the same formal characteristics, as is the case with basketball, the network is said to be *symmetrical*; otherwise it is qualified as *dissymmetrical*: baseball, rounders, the Seven stones, the Deliverance… If the encounter only opposes two players we speak of a «duel of individuals», which can be symmetrical, when the status of the opponents is identical: e.g., fencing, combat sports, tennis, etc., or asymmetrical, when the combatants hold complementary roles: e.g., Quinet, gladiatorial fights…

Basketball is a teams duel whose stable, exclusive, and symmetrical network possesses very determining properties:

• *According to the relationship of Solidarity S*, it is made up of two symmetrical, complete subgraphs of five vertices each called K_5_ “cliques.” Internal cooperation within each team is thus ensured.
• *According to the relationship of Rivalry R*, it consists of a complete K_5_,_5_ “bipartite graph.” The opposition between the two teams is absolute.

The simultaneous consideration of these two relationships S and R determines the bigraph G, which represents the exhaustive support of the relevant motor interactions produced by the two teams (Figure 1). Within the framework of a given federation, this graph is an invariant: it remains strictly identical, whatever the clubs, the composition of the teams or the nations involved may be. If we consider a game other than basketball, the network can remain equivalent, except for the numbers (e.g., football, handball, Prisoners’ base, Dodgeball…), or be structured differently (e.g., Sitting Ball, the *Galine*…). Anyhow, all these motor interaction networks are part of the same equivalence class, according to relationships R and S, and belong to the same class universal.

### A Comparative Approach

Before any quantitative or practical application, a remark must be made: Out of an immense field of possibilities, sport is illustrated on a single, dominant model: the «duel», which covers half of the sociomotor games (e.g., team sports, combat sports, fencing…) at the Olympic games (OG). If we consider some fifteen players, the graph of possible interactions according to the relationships of solidarity and rivalry can take millions of different configurations. Therefore, each game, faced with such a myriad of possibilities, represents a surprisingly deliberate restricted selection that leads us to ask ourselves: What imperatives does this choice abide by?

The fact that this universal of motor interactions can be represented in an objective and verifiable form grants it interesting possibilities in terms of observation and comparison. As a consequence, researchers have a remarkable tool for experimentally exploring the influence of a given motor communication structure, both on group dynamics and the players’ personalities, from an affective, relational perspective. Moreover, beyond those aspects directly observable in the field, the particular structure of this universal adopted by each community can reveal how certain values are embodied and produced. Taking this universal into account, particularly in an intercultural comparative approach, we can shed some light on both the psychological and the sociological levels of groups and individuals.

As we can imagine, the analysis of the properties of the universals in relation to the characteristics of players and communities seems to be of paramount importance. In the studies reported below, we are not interested in the circumstantial characteristics of a particular group of players or a particular game, but in data that test the possible role of universals. Therefore, we will primarily address our comments to the most significant, overall results in this regard.

## Games Under the Light of Observation

This study concerned two groups of 13/14 years old adolescents (13 girls and 13 boys) that took part in a summer camp. It was a piece of *action-research* in the sense of Kurt Lewin, that is to say a study subject to the classical constraints of experimental research while being integrated into the usual daily life of a sleep-away activity centre. The teenagers responded in writing to a sociometric questionnaire before taking part in two games (Sitting ball and Elbow tag), each one bringing together the 26 players, during which all motor interactions were carefully noted down by trained observers.

### The Sitting Ball

In this *each-for-oneself* game any player in possession of the ball can choose on whom to shoot (by direct shot) or to whom to make a pass (by a rebound of the ball on the ground). As a consequence, any player hit by a direct throw must sit down and wait to retrieve the ball without any help before becoming delivered and having the chance to stand up. Such an unusual rule means that players can be expected to be partners (on passing) and opponents (on shooting) at the same time, and the observation of behaviours in the field makes it possible to record who passes to whom and who shoots on whom.

Game-playing went on for 46 min and resulted in 502 motor interactions, including 236 passes and 266 shots. The analysis of the responses revealed that both sociometric and praxic exchanges were dominated by the relationship between the two gendered male/female subgroups. In these intra-sub-group and inter-sub-group interactions, the sociometric responses and the interactive acts were in clear correspondence (*p* < 0.01): overall, the players reproduced on the pitch their on-paper emotional choices and rejections. The network of ludomotor contacts generally embraces the network of friendships and enmities: the praxic is indisputably immersed in the affective.

However, a more detailed examination of the behaviours revealed some contradictory results. Certain subjects who mutually chose each other in their sociometric programs shot at each other during the game; in other cases, two players of the same reciprocal affective dyad interacted sometimes by friendly passes, sometimes by antagonistic shots; besides, a boy ranked 4^th^ in praxic participation was 21^st^ in sociometry. We too found, even in a more accentuated way on the level of motor interactions, the large dispersion of individual statuses that Moreno (1970) calls “socio-dynamic effect” on the sociometric level, but with some discrepancies: the precise comparison of the status of the 26 male and female players in terms of sociometric popularity, on the one hand, and in terms of praxic popularity, on the other hand, revealed a positive correlation at *p* < 0.05 that disappeared at the *p* < 0.01 threshold, which indicates its fragility.

**FIGURE 2:**
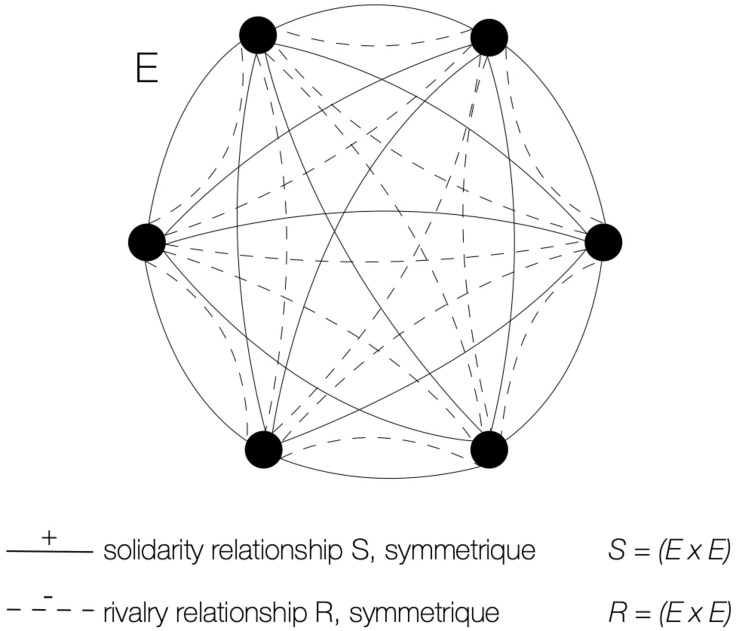
Sitting ball’s universal of motor interactions. Any two pair of vertices are linked at the same time by solidarity relationship S and rivalry relationship R. This ambivalence creates a «paradoxical» game.

As far as the universal of the motor interactions is concerned, the Sitting ball is very different from basketball (Figure 2): while the latter splits up the players into a couple of antagonistic blocks, the former condenses them into a single, complete graph for each of the two relationships of rivalry R and solidarity S. It follows that, in terms of internal logic, all the players are both partners and adversaries: a participant who has just addressed a friendly pass to a comrade can suddenly become the antagonistic target for this same comrade, who chooses *for sport* to put him temporarily out of the game. This turnaround is very badly received by a victim who usually shouts out of treason. However, this gesture of transgression of a tacit connivance is authorised by the internal logic of the game: each participant has complete freedom to choose with regard to the same player between the solidarity pass or the rivalry shot. The Sitting ball is therefore placed under the sign of ambivalence, which results very shocking in relation to usual morality because all participants are simultaneously allies and enemies. For this reason, we will speak of *paradoxical* games since it is indeed the relational structure of the universal of communications that holds the key: it is this which offers the possibilities of acting in such and such a way, which conditions the decisions of the players by granting them more or less of leeway. Furthermore, the inconsistencies identified in the results depend directly on it.

The observation of the behaviours of the players revealed another phenomenon. The constraints of space, distance, and movement linked to the circulation of the ball caused unexpected, unforeseen proximities. Suddenly, a player receives the ball and finds herself face to face with a comrade toward whom she has hardly any acquaintance. Often times, in the heat of the moment, she will interact with him in what sometimes is the start of a chain of unexpected, subsequent connections. Individuals who ignore each other in everyday life find themselves surprisingly side by side and cannot help but interact. These unplanned interactions can pull a relational trigger and create new bonds among the participants. In the affective and relational field, an imposing research involving hundreds of practitioners, led by Lavega et al. (2013) with the help of many other researchers, has shown how pronounced the impact exerted by traditional games can be, in particular by multiplying feelings of pleasure and solidarity: Game-playing is not only the carbon-copy of a completely pre-determining affective network, but the source of interesting educational extensions, thanks to its capability of sparking new relational interactions.

### Elbow Tag

As we said, our objective is to test the differential influence of some of the possible forms of the universal of motor interactions. The praxic data collected during a sequence of Elbow tag were compared to the sociometric responses, all along the same players as before. At the start of this game, 24 of the total 26 participants are divided into a large circle of couples clinging by each other’s elbow; the other two are designated «hunter» (it) and «hare» (runner). Pursued by the hunter, a hare finds its salvation by *hooking* to the free arm of one of the two players of any of the couples in the circle; in that very moment the other player of the standing pair gets released and becomes the new hare, which runs away at full speed before clinging in turn to one of the waiting pairs. If the hare is touched before hanging on there is a swap of roles between the hare and the hunter. The most popular role among players is the hare because it allows a lot of fantasy, tricks, and feints: some hares hang on after only a few seconds, while others prolong their facetious provocations for several minutes.

The 26 previous players took part in a game that lasted 42 min with 130 clinging motor interactions. The universal of communications is identical to that of the Sitting ball (Figure 2). However, in this game of *each-for-oneself* without pre-established teams, the internal logic of the universal imposes an original operating mechanism. The interactive process at the heart of the game is relatively complex. It is apparently a binary relation –the hare clings to a comrade –, but the relationship is ternary in reality: by their attachment, the hare has freed the second player of the pair, who becomes the new hare in accordance to the universal of sociomotor roles. Sometimes the running hare even directs its course so that the *hare-to-be* standing player will be easily struck by the hunter when the role-change occurs, which creates a quaternary relationship. These phenomena are not a mere speculation on the observers’ part: the teenagers express them loudly by their exclamations on the spot and by their comments after the game.

The chain of praxic actions of hooking and unhooking revealed that, here again, we find a global correspondence between the sociometric links according to the male/female subgroups and the network of motor interactions. Still, at the individual level, the correlation between the sociometric and praxic statuses observed at the Sitting ball was broken. The ludomotor exchanges no longer corresponded trustworthily to the socio-affective attractions, and the playing mechanism of the game caused incongruous interactions, usually refused elsewhere. In the same vein, the two scales of praxic popularity established for each of the two games did not show any significant correlation. The account is clear: The universality of communications generates relationships specific to each game, creating new contacts and opening up a field favourable to new attractions.

However, the mechanism of the universal led to bizarre results, intriguing and apparently impossible to explain. For example, one of the players with lower sociometric status was surprisingly the one that his comrades unhooked most often, getting therefore the highest participation rate. What was going on? After a first release, this player systematically clung to a comrade very appreciated by the group who, because of this popularity, was quickly hooked by a hare, an action that *ipso facto* freed our sociometrical neglected player giving him the chance to prance and twirl around the couples and recommence his strategy of selective hooking. This ternary relationship, the source of the adventures of the game, leads us to an obvious conclusion: We can only understand the alchemy of the relationships between players by taking into account the mechanisms of the development of the motor interactions of the universal.

### Hens-Vipers-Foxes

**FIGURE 3:**
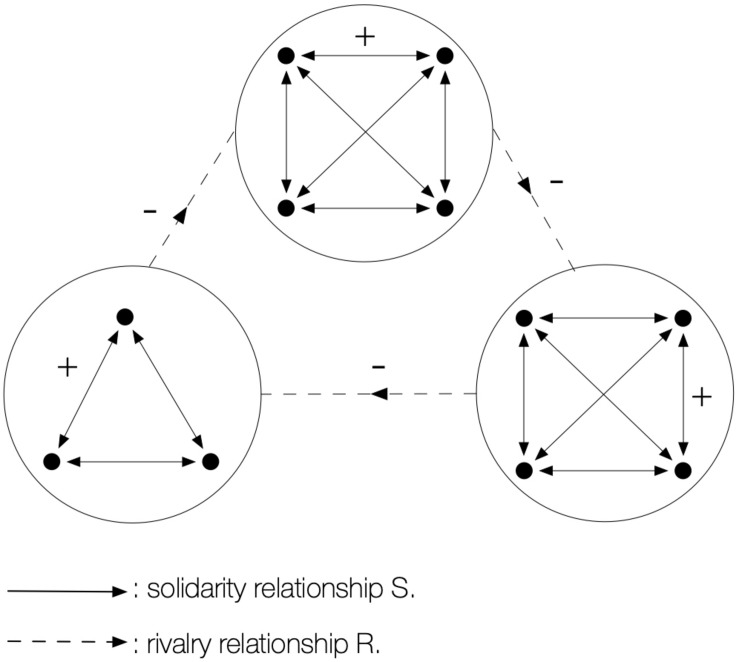
Hens-Vipers-Foxes game’s universal of motor interactions. Three (solidarity) cliques are situated on an (intransitive) circuit of rivalry.

In Sitting ball ambivalence depends on the individual decisions of players, who have the option of choosing whether or not to be *uncoherent*. On the other hand, in Hens-vipers-foxes it is the game itself, by its internal logic, which inevitably imposes the discordance no matter how much players like it or not. This game puts three teams in opposition, each one with a separate camp: *hens*, *vipers*, and *foxes*. The confrontation is carried out by a relation of tagging by simple touch: hens can tag vipers; vipers can take on foxes; and foxes can capture hens (Figure 3). A tagged player becomes a prisoner and taken to the camp of their predators, and prisoners can be delivered by one of their partners with a simple touch.

**FIGURE 4:**
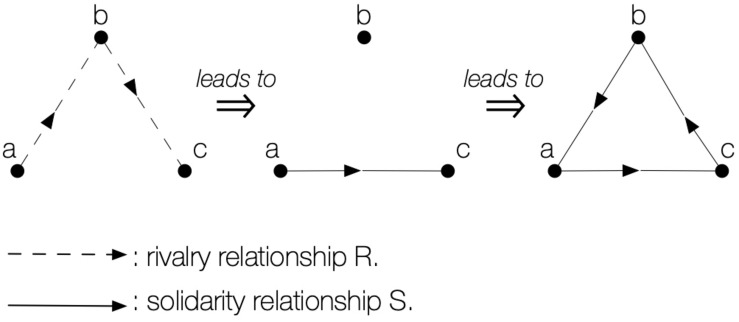
Mechanism of reversal of relationships that makes part of the internal logic of Hens-Vipers-Foxes. The fact that player «a» can tag «b» (who is a menace for «c») protects «c» from the attacks of «b»; thus, if «a» is captured «c» has no defence from «b». Therefore, «c» will be reluctant to tag their designated adversaries for they are their paradoxical protectors.

The logic of this game is disturbing: When a hen captures a viper deprives itself of an agent who protects it from the fox! The more the players get the impression of winning by accumulating prisoners, the more they deprive themselves of their only defenders, and the more they contribute to their own downfall. As shown in Figure 4, the motor action of the players who capture an opponent paradoxically becomes the germ of their defeat! Ambivalence is therefore absolute and ineluctable here, and leads to original conducts in the field: hesitation, negotiations, alliances, betrayals, and reprisals. By means of a mechanism as simple as the grid of its motor interactions, the universal of this game creates an unusual relational world and causes destabilising interactions which force participants to think twice about their conduct regarding the others.

### A Universal Source of Creativity

We can put together the content of the previous observations in a few lines:

• The players’ praxic exchanges are globally in deep correspondence with their socio-emotional links.
• However, relational creativity can be provoked in game situations by generating new relationships likely to create future contacts.
• Some traditional games feature ambivalent relationships which give rise to paradoxical discrepancies that put the usual relationships upside down.
• In *each-for-oneself* games, any player, not subject to any team pressure, can choose at will who will be partner and who will be opponent: everyone is the master of their motor conducts and decisions.
• In conclusion, the experiences generated by each traditional game vary considerably depending on the internal logic of the considered universal.

By arousing great variations in behaviour, these characteristics can be rich in educational implications: creation of new relationships, disconcerting contacts that prepare for social adaptability, embodiment of individual power of initiative and decision. The numerical results recorded during these games reveal important phenomena, but they cannot be generalised in their detail: They serve to detect the most revealing points and to guide future research. Basically, they reveal the crucial role of the universal of motor interactions in the development of sports games and its effects on the conducts of players, regardless of their individual characteristics.

## The Major Structures of the Universal of Communications

### A Plurality of Models

After processing multiple datasets, it has been possible to identify the different ways in which the universal of motor interactions is present in all the sociomotor games. These are the main models we identified:

• *The duels*:
  – *duel of individuals*:
    • *symmetrical*: boxing, fencing, tennis, cane, stick.
    • *dissymmetrical*: *Quinet^1^*.
  – *duel of teams*:
    • *symmetrical*: football, hockey, Prisoners’ base^2^, Stealing sticks^3^.
    • *dissymmetrical*: Cops and robbers, Seven stones^4^, Capture the flag (one flag version) …
  • *Measured-performance races*: athletics, swimming, rowing, sailing.
  • *Cooperative or semi-cooperative games*: canoeing, team rowing, acro-sport.
  • *Games with an original structure*:
    – *Each-for-oneself*: the Sitting ball, Elbow tag, the *Galine^5^*.
    – *A-team-against-others*: the Fishing net^6^, Octopus tag^7^.
    – *One-against-all*: the Gouret^8^, the British bulldogs.
    – *Circular confrontation between coalitions*: Hens-vipers-foxes.

Some other structural traits could be added, especially those which lead to drastic modifications of the internal logic of the games and give way to paradoxical relationships or the reversal of alliances, but the scope of this paper does not require so.

### Underlying Cultural Correspondences?

Does this structural diversity, brought to light in an objective way, allow any interesting cultural interpretations? In fact, once we are aware of such a praxic variability, there is no reason to keep considering the game as a *black box* whose output would be indifferent with regard to the functioning mechanisms of the activity. In each case a *game grammar* linked to the internal logic of the game operates and selectively predetermines certain constants and sequences. For this reason, it is necessary to bring to light the operational processes specific to the universal of each category of games to infer the most probable relational and social effects.

Traditional games are the fruit of a history that has shaped their structures according to the values and collective representations of each region. So, we can expect that universals be in the image of the culture they belong to: Games’ morphology entails cultural meaning. In this sense, it seems possible to draw up a *ludoscopy* of the different sporting games to uncover those fundamental structures that unveil major social trends. It is not frivolous to say that corporal practices, ludic practices in particular, plant the seeds of future social behaviours. In the same way parents like to find in the younger generations ways of acting and reacting in agreement with their conception of adulthood, sporting games install certain predispositions to future conducts.

This study suggests the hypothesis that the functioning structures of sporting games support underlying values and categories of action that predispose participants to forge their relationship with others in the suggested way. Even though, based on the main features of these structures and without indulging in uncontrollable speculation, can we offer any stimulating interpretations of the aspirations and values staged by the corresponding societies?

### The Supremacy of Duels and the “Reversal Effect” of Sport

Consider the OG, which are meant to be representative of the major sporting games on the planet. The figure is impressive: 50% of the sociomotor events of the Rio 2016 games were duels, both between individuals or teams. This highly majority percentage of duels suggests that this binary competitive model is today in accordance with the main collective representations of host cultures.

The structure of this binary confrontation, as illustrated by the graph of the universal of motor interactions in Figure 1, is remarkably clear: The ambivalence of the relational paradox, the *betrayal* of the sudden reversal of alliances are all totally unknown. As we have noted, the properties of exclusivity and stability of the relationships R and S make it possible to systematically declare and praise a final winner, according to the terms stablished by the universal of scores. Every duel ends with the absolute domination of one of the two players or super-players, to the detriment of the unfortunate loser.

However, if the champion’s victory is to be indisputably glorified, the conditions must be equal in the first place. In order to balance the chances of success for each opponent, the universal is symmetrically composed by two “cliques” of the same size opposed by a “complete bipartite subgraph” (Figure 1). Sports competition, ostentatiously egalitarian at the start (equality in equipment, team numbers, age categories, weight….), will end with a systematically unequal outcome at the finish. In other words, the equality of opportunities, so frequently advanced as a factor of consensus and equity, is conversely what will legitimise the eventual domination by fracturing the relations between practitioners. In the end, equality is at the service of inequality: This is the “reversal effect” of sport.

This rapid analysis of sport’s vindication of performance and domination with regard to others suggests, on one hand, that contemporary societies push to the front a conquering and domineering elite. On the other hand, the confrontation of two united clans –ultimately reduced to a single player – offers an easily decodable spectacle, favourable to the projection of the passions of the spectators. The duels’ model, exemplary clear and fertile of uncertain results, possesses the limpid and objective characteristics of a very valuable mass spectacle, both tactically and emotionally speaking. From this perspective, sport is an incontestable success.

We should note, however, that this reversal effect, this harsh, aggressive reality of the effects of domination, is very difficult to reconcile with the declared objectives of fraternity. Sports’ remarkable conditions to become a mass spectacle are not systematically compatible with an ethical, educational perspective oriented toward humanism and solidarity yet advocated by international governing bodies, what acutely poses one true problem as far as sports culture is concerned.

### A Homogeneous Methodological Approach

The universal of motor interactions, a tool for observing and analysing games behaviours, can be used for intracultural interpretations as well as for intercultural comparisons. Playing games socialises the children, and thereby predisposes them to the influence of the structures and habits of their environment. Several ethnological pieces of research have already studied games by describing and classifying them, sometimes examining their connection with the norms and values of their host society (Griaule, 1938; Charles, 1955). Speaking of the Dogon child, Marcel Griaule does not hesitate to write: “Through games, they prepare themselves in their own way for the struggle that awaits them,” through games that “constitute a kind of introduction to cultural life.” This is the core of our project: Can universals unveil the mystery of the deep, often unnoticed phenomena of play? Can the objective characteristics of the relational networks of sporting games and their operating mechanisms shed any thought-provoking light on the content of the cultures in which they flourish?

From an anthropological perspective, Roberts et al. (1959) proposed a classification of the games of fifty ethnic groups by matching their ludic practices with some social options: strategy, chance, physical skill. However interesting the authors’ conclusions may be, this kind of research does not take into account specific, essential characteristics of the games, particularly those of physical activities’, which are in total more than the double of the rest of practices. Conceptual unity and methodological homogeneity seem basic at first if we want to grant games full status as scientific reality. This is the point of view defended by many researchers who, for the past 40 years, have taken games as their research object from the angle of the bodily conducts and motor action requested. We are going to rely on these works, which have studied the universals of many games and in particular the ups and downs of the universal of motor interactions.

In this spirit, we have brought together sociomotor games from many regions, games identified and studied meticulously by many researchers: Ferretti (2016) for Ticino (Switzerland); Bouzid (2000) for the Kerkennah archipelago (Tunisia); Staccioli (2004) for the games of Basile (Italy); Ould Saleck (1994) for Mali and five other neighbouring countries (Senegal, Burkina Faso, Mauritania, Côte d’Ivoire, and Niger); and ourselves for the illuminations of Ango (2010) (Parlebas, 2010), the children’s games painted by Brueghel (2003) (Parlebas, 2003), the prints by Prévost (2017) (Parlebas and Depaulis, 2017), and Jacques Stella’s drawings (1998) (Parlebas, 1998). We thus have sets of games from different countries that enable unprecedented intercultural comparisons.

This comparative approach allows many possibilities for contrasting games from the same region at different times, games from the same period in different regions or games from different regions and eras. Table 1 groups together the sociomotor games of the regions mentioned above, listing the games at periods that spread over time. Besides, the activities are categorised according to the four main sections that we have previously identified: duels, races, cooperative or semi-cooperative games, and games with original structures.

**TABLE 1:** Distribution of the different models of the universal of motor interactions according to different countries and epochs (percentages of respective sociomotor games).

Games categories					
Games corpora	Duels	Measured racing performances	Cooperative or semi-cooperative games	Originally structured games	Total
ANGO (1525) *n* = 158	9%	/	**64%**	27%	100%
BRUEGHEL (1560) *n* = 33	33	/	30	37	”
PRÉVOST (1587) *n* = 144	27	/	30	43	”
BASILE (1625) *n* = 28	29	/	18	**53**	”
STELLA (1657) *n* = 84	36	/	43	21	”
TESSIN-SWITZERLAND (1900) *n* = 33	20	/	42	38	”
MALI (1990) *n* = 128	37	/	2	**61**	”
AFRICAN COUNTRIES (1990) *n* = 275	38	/	3	**59**	”
TUNICE (1995) *n* = 240	29	/	32	39	”
2016′ OLYMPICS Rio de Janeiro *n* = 182	**50%**	25%	25%	/	100%

*The universal’s forms are distributed with exuberant diversity, a testament to how different cultural representations can be in regard to social linkage. Bold values are indicate the most prominent values in some of the datasets.*

## An Ethnomotricity That Holds an Identity

What does Table 1 reveal? Certainly, a strong international diversity in the universal of communications. Each cultural area takes pleasure in developing a ludic heritage distinct from that of the others. We can advance the term ethnomotricity insofar as any region cultivates its own ways of using body gestures to play, to maintain links of solidarity and opposition that belong to tradition and terroir. This ethnomotricity reveals the search for the affirmation of an identity that, through frequent references to its *roots*, constitutes an original ludic heritage that goes far beyond the simple diversity in the naming of games or nuances of detail: It deals with the deep dissimilarities between the networks and processes of motor interaction which connect players to each other. What are the main interpretations that can be inferred from Table 1?

### The Olympic Games

**FIGURE 5:**
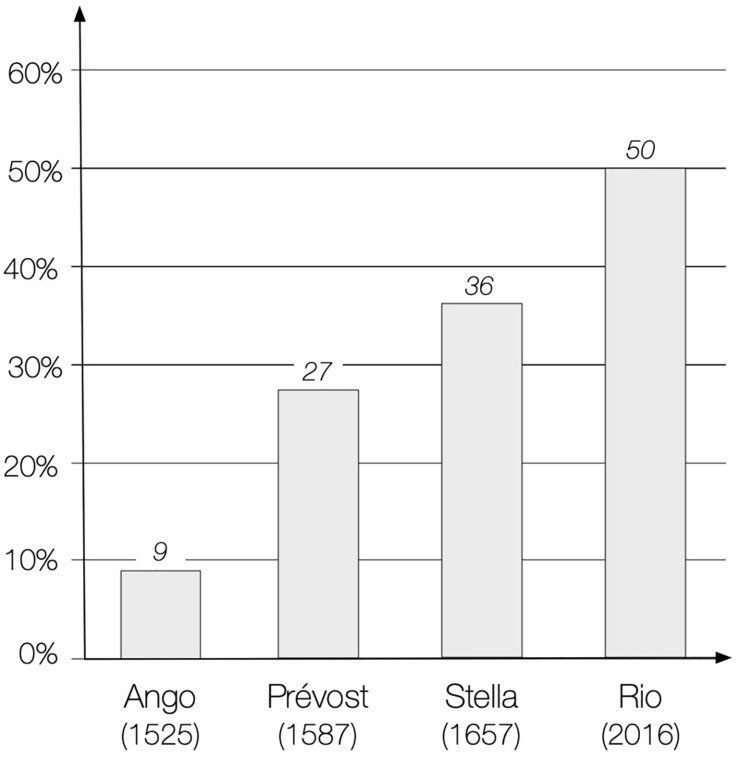
Percentages of duels within sociomotor games. After an evaluation of games corpora from the Renaissance to nowadays, it shows the spectacular of the progressive increase of the proportion of duels. The duel structure has prevailed little by little becoming the dominant model of sports today.
• The OG are the only ones to have “races”, sanctioned by precise measurements of time or space, calling for peak performances and world records.
• The OG are the only ones to present no universal of original motor interactions.

Nowadays a set of games from around the world honoured and supported by more States than the UN contains, the OG can serve as a baseline for immediate comparison:

• Among the sociomotor games, the OG show a very high percentage of duels (50%), much higher than that of all the other categories. In our corpus this percentage gradually increases from the 16th century to the present day (Figure 5).

The verdict is clear: The OG, which symbolise what modern physical games are, have frankly broken with traditional games, being defined by highly specific traits linked to a culture of excellence in antagonism and to the search for outperformance.

### On a General Level

**FIGURE 6:**
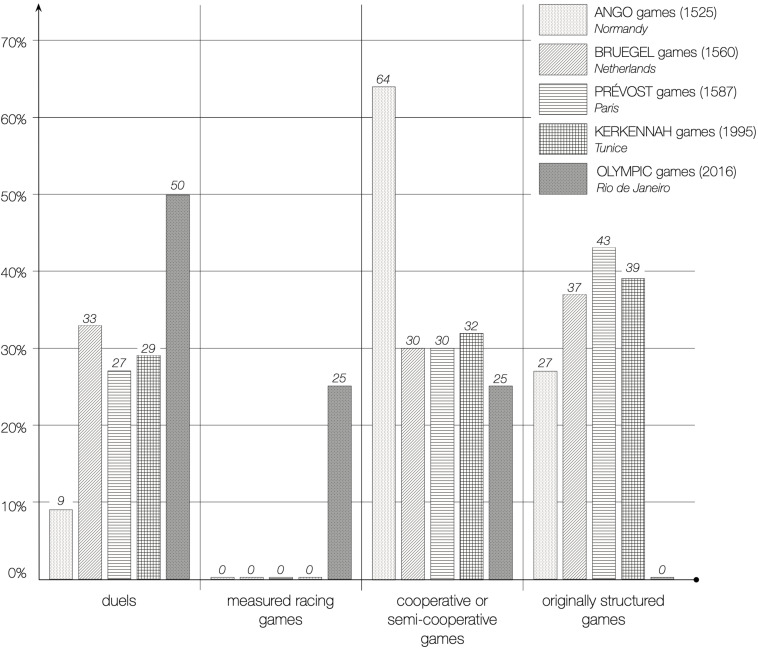
Distribution of the main ludic categories in several cultural areas. The distribution of the categories of the universal of motor interactions models is very variable in regard to different cultures. Along with the clear differences between Ango and Rio, separated by five centuries, there some similarities to be explored among Bruegel, Prévost and Kerkennah.

Bearing in mind what we have just learnt about sports, it is possible to understand how universals contribute to the study and understanding of sporting games by thoroughly looking into Table 1:

• Most communities, including the OG, but excluding African countries, give a significant place, of the order of a quarter, to cooperative or semi-cooperative games. The reality of motor cooperation is therefore present in the majority of the cultures here considered.
• The proportion of traditional games with original universals is abundant, sometimes even in excess of 50% of the cases. This is one of the indications of an intense social creativity in the search for infrequent, stimulating, destabilising situations that demand a strong individual adaptability to unusual activities. The equality offered by traditional games is not attached to measurement and performance as in sports. Instead, taking into account the constraints of internal logic, this sought-after equality is part of the free choice offered to each player to behave according to their emotional and relational preferences.
• Belonging to the same region is not enough to standardise the modalities of the universal. Although they belong to the same extended cultural area (Normandy and the Parisian region), Ango games appear very different from Prévost and Stella games (Figure 6). The social categories of belonging (rural and urban) as well as the corresponding lifestyles have a considerable influence on the ways of experiencing one’s body and the others’ bodies during games. The frequency of farm work practiced in community, which requires a great solidarity in the accomplishment of the tasks, can be put in correspondence with the abundance of the cooperative games of the illuminations of Ango (64%).

### Dissimilarities and Similarities

As a whole, these results represent a kind of survey that offers a partial but solidly documented picture of ancient and modern ludic practices in different countries, pointing at remarkable observations that well deserve a deeper look:

• Alongside the two very different extreme corpora: Ango (Normandy around 1525) and Rio (the planet, 2016), undeniable similarities appear between Brueghel (Netherlands, 1560), Prévost (Paris, 1587), and Kerkennah (Tunisia, 1995) sets, yet far apart in space and time (Table 1). We can hypothessze that the island of Kerkennah, being located an hour by boat from the Tunisian coast, has been somewhat protected from sporting influences and has kept its traditional recreational roots. What all this work shows is the weight of the social and geographic contexts of belonging, the representations and the imagination of host cultures. We are indeed in the presence of an *ethnoludism* that marks with its cultural imprint the heritage of the games.
• Mali and certain African countries present aspects which contrast with the previous results (Table 1 and Figure 6). During a prolonged field survey, Ould Saleck (1994) analysed data relating to Mali (128 games) and five other neighbouring African countries (275 games including those from Mali). The results are spectacular: high percentage of duels (37% and 38%), virtual absence of cooperative games (2% and 3%), total absence of racing games, and proliferation of games with an original structure (61% and 59%). This abundance of original games is localised in the models *one-against-all* (76% of these games) and *each-for-oneself* (19% of these games). Obviously, Malians and their neighbours appreciate individual prowess and are adept at fighting and confrontation.
• The comparison between the OG and the African games is very enlightening (Table 2) in regard to duels. African games favour team duels (76% of duels) while OG give the lion’s share to individual duels (74%). Besides, unlike African games, which give clear priority to mismatched duels (63%), the OG are radicalised by completely excluding any asymmetric duel. Equality wants to be the standard-bearer. Spectacularly, the reversal effect of duels gets to the peak in sports the Olympic way.

**TABLE 2:** Olympic games and African games.

Corpora		
Duels	Olympic games *n* = 93	African games *n* = 105
Individual duels	**75%**	24%
Team duels	25%	**76%**

Total	100%	100%

Symmetric	**100%**	37%
Dissymmetric	/	**63%**

Total	100%	100%

*Team duels and dissymmetric duels are clearly majority in African games, just the opposite to the Olympic duels, which highlight the individual and the equality of arms. Bold values are indicate the most prominent values in some of the datasets.*

## The Originality of Traditional Games

Any in-depth study would require to immerse the previous data back into the social and historical environment they come from. It would also be advisable to increase the number of corpora from very different countries. However, the results obtained here are sufficient to offer interesting interpretations, which can serve as indicators for subsequent studies:

• Ludodiversity is a confirmed phenomenon: The universal studied above has structures that can be extremely different from one game to another, and this rich patrimony should not be condemned in the name of any demand for standardisation. The adoption of the same sports structures on a world scale favours to a certain extent the relations between foreign practitioners, but at the risk of the abolition of regional identities. Globalisation should not be opposed to the recognition of the local originalities of the regions. As Levi-Strauss wrote, it is necessary to respect an “optimum of diversity” (1983), and the identity of a culture is also played out in its games.
• The analysis of universals reveals that the alleged superior complexity of sports is an illusion. Quite on the contrary, what is evident is the remarkable simplicity of symmetrical duels and races, in contrast to the lush palette of the original structures of traditional games. These are often presented as *little* inferior games, just *preparatory to team sports*. However, it is simply not true that traditional games be at the beginning of a linear scale of complexity culminating in the higher echelon of sport, thus seen as the crowning achievement: Between traditional games and sports there is not a difference in degree, but in nature.

This type of exploratory analysis on the operational mechanisms that support the development of sporting games has been extended to all universals, and has shown the interlinks that connect them to each other. The universals of scores, sociomotor roles, and sub-roles confirm the inquiry possibilities offered by the universals of motor interactions. This works reveal that sporting games constitute a way of forging relationships with others, of making decisions and of constructing categories of action that create predispositions to future behaviours: A way of experiencing the world simply put.

Issued from the rules of the games, universals represent different social frameworks that call for individual motor action according to more or less permissive action logics. Taking them into account makes it possible to link the carefully collected empirical materials to a reflection on the foundations of ludic conducts and, above all, on the foundations of motor action in general. In brief, the conclusion suggested by this study is that sporting games represent a specific world, symbolised by the presence of a system of universals, rich in psychological and sociological elements, that calls out for in-depth scientific research.

## Author Contributions

The author confirms being the sole contributor of this work and has approved it for publication.

## Conflict of Interest

The authors declare that the research was conducted in the absence of any commercial or financial relationships that could be construed as a potential conflict of interest.

## Footnotes

1 One player with a stick tries to hit away as far as possible a little piece of wood, the *quinet* itself, while the other one tries to catch it to become the hitter.

2 Also known as *Prisoners’ bar*, this is a tagging game between two equal teams in which a player can only by captured by those other team’s players leaving their base *after* the opponents to be caught.

3 The players from two equal teams try to steal a certain amount of sticks, or stones, from each other avoiding to be temporary put out of play if tagged while on the other team’s half-court.

4 After knocking down the *seven stones* tower made by team A, team B players try to capture all A players hitting them with a ball before they rebuild up the tower.

5 Intriguing tagging game in which enemies are declared out loud, pebbles aimed and thrown at the *galine* to put them in prison, and everybody tries to get as many pitching opportunities as possible avoiding to get caught after the *galine* is hit.

6 A closed, circular net of fisher-players hold hands up until the count gets to their secret number; in this moment the arms are pulled down closing *the net* and trying to catch as many fish as possible; captured fish become fishers.

7 Free players run from one end to the other trying to be safe from the octopus, having to sit where caught and help the octopus catch the rest of fish by waving their arms.

8 Free players stay safe while having their sticks in their assigned holes, which draw a semicircle with a bigger, common hole in the centre; “it” tries to get the *gouret*, a little hard ball, into any player’s hole forcing a role change; “it” also gets free if taking an empty hole with the stick; free players try to hit the *gouret* away and stay safe at their own holes or taking any momentary empty hole.
